# Peripheral Blood Mononuclear Cell Oxygen Consumption and Systemic Bioenergetics in Glaucoma Management

**DOI:** 10.3390/ijms27062704

**Published:** 2026-03-16

**Authors:** Chun Hsiung, Ta-Hung Chiu, Wei-Ting Yen, Da-Wen Lu

**Affiliations:** 1 Department of Ophthalmology, Tri-Service General Hospital, Taipei 114202, Taiwan; drchunhsiung@gmail.com; 2 Department of Ophthalmology, College of Medicine, National Defense Medical University, Taipei 114202, Taiwan; 3 Department of General Medicine, Tri-Service General Hospital, National Defense Medical University, Taipei 114202, Taiwan

**Keywords:** glaucoma, mitochondrial dysfunction, peripheral blood mononuclear cell, oxygen consumption, neuroprotection, neurodegeneration, oxidative stress, retinal ganglion cells

## Abstract

Glaucoma is a multifaceted optic neuropathy, characterized by the progressive loss of retinal ganglion cells. This damage frequently continues even after intraocular pressure (IOP) has been effectively lowered. This resistance to conventional IOP-lowering therapy underscores the critical role of interacting IOP-independent mechanisms; specifically metabolic failure and systemic mitochondrial dysfunction have emerged as key parallel drivers. This review analyzes the paradigm shift from a pressure-centric model to a bioenergetic one, focusing on mitochondrial function, peripheral blood mononuclear cell (PBMC) biomarkers, and oxygen consumption dynamics. We synthesize evidence demonstrating that glaucoma patients exhibit a metabolic vulnerability, characterized by lower PBMC oxygen consumption rates and depleted systemic nicotinamide adenine dinucleotide levels relative to healthy individuals. Furthermore, compromised systemic respiratory performance correlates with more rapid worsening of visual fields and structural thinning, independent of IOP status. Moreover, we delineate the role of Complex I defects, SARM1-mediated axonal degeneration, and proteomic alterations, which indicate defective mitophagy. These findings establish systemic metabolic profiling as a valuable supplementary tool for assessing patient risk and support the clinical translation of neuroprotective therapies targeting mitochondrial bioenergetics, specifically nicotinamide, pyruvate, coenzyme Q_10_, and metformin.

## 1. Introduction

Glaucoma is a complex, multifactorial optic neuropathy defined by the progressive degeneration of retinal ganglion cells (RGCs); it remains the leading cause of irreversible blindness worldwide [1,2]. Generally, the management of primary open-angle glaucoma (POAG) has been predicated on a paradigm centered on ocular hypertension (OHT). This framework posits that increased intraocular pressure (IOP) serves as the primary modifiable risk factor driving mechanical strain on the lamina cribrosa (LC) and resulting in retinal ganglion cell apoptosis [3,4]. While the reduction in IOP through pharmacological or surgical means remains the only proven therapeutic intervention, clinical outcomes frequently deviate from this pressure-centric model.

A substantial proportion of patients continue to experience worsening visual fields even after achieving target IOP goals. Normal tension glaucoma (NTG), characterized by optic neuropathy in the absence of statistically elevated IOP, represents a significant global disease burden. Recent epidemiological data indicate that NTG constitutes 83% of all open-angle glaucoma cases, a prevalence pattern that is particularly pronounced in Asian populations [5,6]. Conversely, the phenomenon of OHT presents a biological paradox wherein individuals sustain elevated IOP for decades without developing glaucomatous optic neuropathy [7]. 

This clinical disparity between neurodegeneration at normal pressure and neuronal survival at high pressure compels a fundamental re-evaluation of the disease etiology. It suggests that IOP is not the sole determinant of RGC survival but functions as a stressor that interacts with the intrinsic susceptibility of the individual [8,9]. Emerging research has identified metabolic vulnerability and mitochondrial dysfunction as the primary interacting drivers of this intrinsic susceptibility [8,10].

Operationally, we define metabolic vulnerability as a state of systemic bioenergetic fragility characterized by quantitative deviations from healthy cohorts: specifically, reduced basal peripheral blood mononuclear cell (PBMC) oxygen consumption rates (OCR), systemic nicotinamide adenine dinucleotide (NAD^+^) depletion, and decreased peripheral mitochondrial DNA (mtDNA) copy numbers [9,11]. Rather than acting in isolation, these bioenergetic deficits operate in parallel with vascular dysregulation, neuroinflammation, and biomechanical strain, collectively lowering the threshold at which ocular tissues can withstand physiological stress. To support their extreme metabolic demands, the retina and optic nerve rely on neuroglobin concentrations nearly 100 times higher than those in the brain [12,13]. Furthermore, this protein is further upregulated in advanced glaucoma to protect against hypoxic, ischemic, and oxidative stress [14]. Consequently, any defect in mitochondrial respiratory function, specifically within the mitochondrial electron transport chain (ETC), renders these tissues acutely susceptible to physiological stress that would otherwise be benign.

In this narrative review, we consolidate recent findings regarding the mitochondrial theory of glaucoma, which marks a shift from pressure-lowering strategies to bioenergetic medicine. We examine the prognostic utility of PBMC OCR and systemic NAD^+^ levels as clinical biomarkers for disease progression. Furthermore, we delineate the molecular mechanisms of the sterile alpha and TIR motif-containing 1 (SARM1) protein-mediated degeneration and evaluate the therapeutic promise of metabolic enhancers currently under investigation. It has been demonstrated that neurosteroids may improve the metabolic state of surviving retinal cells after ischemic injury, suggesting a potential use for them in glaucoma [15]. We build upon these neuroprotective concepts by focusing on established targets such as NAD^+^ precursors and pyruvate, as well as emerging candidates including HDAP2, coenzyme Q_10_, and metformin.

## 2. Search Strategy

PubMed, MEDLINE, and Embase were searched (1 January 2000 to 31 December 2025) using keyword combinations including ‘glaucoma’, ‘mitochondrial dysfunction’, ‘peripheral blood mononuclear cell’, ‘oxygen consumption’, ‘neuroprotection’, ‘oxidative stress’, and ‘retinal ganglion cells’. Emphasis was placed on recent literature detailing systemic metabolic profiling and emerging clinical trials, while retaining foundational context regarding intraocular pressure paradigms.

## 3. The Mitochondrial Theory of Glaucomatous Neurodegeneration

The pathophysiology of glaucoma has evolved from a purely pressure-dependent model into a multifactorial framework where metabolic insufficiency acts as a significant parallel driver [9]. To ensure mechanistic clarity, we categorize this bioenergetic decline into three progressive parameters:

- Mitochondrial dysfunction: upstream structural and enzymatic defects, primarily reduced Complex I activity, mtDNA mutations, and reactive oxygen species (ROS) leakage [16].
- Metabolic insufficiency: the functional consequence, defined by diminished spare respiratory capacity (SRC) and restricted ATP scaling.
- Bioenergetic crisis: the late-stage pathological cascade characterized by profound NAD^+^ depletion, collapsed ATP pools, SARM1-mediated axonal degeneration, and abortive autophagy [17].

This intrinsic metabolic deficit renders ocular tissues unable to withstand concurrent physiological stresses such as IOP fluctuations or ischemic events, that might otherwise be benign [9,18].

### 3.1. Ocular Tissue Vulnerability

The vulnerability of ocular tissues in glaucoma is dictated by their metabolic requirements. The optic nerve head (ONH) represents a critical bioenergetic transition zone, where the convergence of unmyelinated axons imposes an exceptional metabolic demand [19].

#### 3.1.1. Trabecular Meshwork (TM)

The trabecular meshwork (TM) is a metabolically active tissue responsible for regulating aqueous humor outflow. Maintaining the contractile tone of the TM and remodeling the extracellular matrix (ECM) requires significant ATP turnover [20]. In POAG, TM cells exhibit a distinct mitochondrial phenotype. Izzotti et al. have reported the accumulation of oxidative DNA damage in glaucomatous TM cells, particularly 8-hydroxy-2-deoxyguanosine (8-OHdG), and the presence of the pathogenic 4977 bp common deletion in mtDNA [21,22].

This large-scale deletion eliminates genes encoding crucial subunits of the ETC, which leads to bioenergetic failure, collapsed membrane potential, and cellular senescence [20,23]. The resulting energy deficit impairs the ability of the TM to clear debris and maintain cellularity, leading to the fusion of trabecular beams and the subsequent elevation of IOP [20,22]. Thus, mitochondrial dysfunction in the TM acts as a primary instigator of the OHT that characterizes high-tension glaucoma (HTG) rather than a secondary effect [22,24].

#### 3.1.2. Lamina Cribrosa (LC) Cells

The LC acts as a support structure for RGC axons exiting the eye. LC cells from glaucoma donors display severe metabolic defects, including a collapsed mitochondrial membrane potential, calcium dysregulation, and reduced ATP production [25]. These cells generate elevated basal levels of ROS, which create a pro-inflammatory environment and impair the ECM remodeling necessary to preserve the architectural integrity of the ONH [26,27,28]. Concurrent metabolic failure acts in concert with inherent biomechanical strain, severely impairing the cellular remodeling necessary for LC structural maintenance and contributing to the clinical cupping phenotype [7,26,29].

#### 3.1.3. Selective Vulnerability of RGCs: The Bioenergetic Tipping Point

RGCs are uniquely vulnerable due to their morphology and physiology. The intraocular segment of the RGC axon is unmyelinated to maintain optical transparency. This unmyelinated segment relies on continuous rather than saltatory conduction, which is approximately tenfold less energy-efficient and necessitates a high density of mitochondria, voltage-gated sodium channels, and Na^+^/K^+^-ATPase to sustain ionic homeostasis [19,30,31]. Given the spatial separation between the laminar axon and the soma, mitochondrial biogenesis occurs primarily in the cell body and necessitates active anterograde axonal transport to sustain the exceptional bioenergetic demands of this unmyelinated segment [32].

### 3.2. Mitochondrial Bioenergetics: Electron Transport Chain (ETC) and Complex I Defect

**Figure 1:**
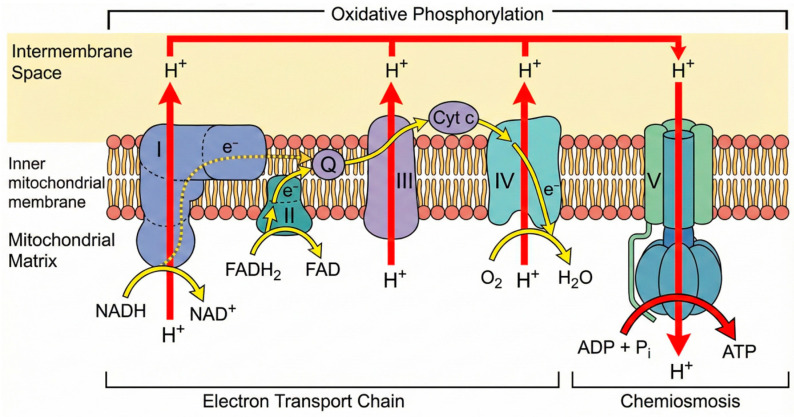
Schematic of the oxidative phosphorylation system and mitochondrial electron transport chain (ETC). The ETC is embedded within the inner mitochondrial membrane, including two mobile carriers, coenzyme Q_10_ (Q) and cytochrome c (Cyt c), and four multiprotein units (Complexes I–IV). The generated proton motive force powers the return of protons to the matrix via ATP synthase (Complex V), enabling ADP phosphorylation to produce ATP. Red arrows indicate the direction of proton (H^+^) movement across the membrane, and yellow arrows represent the path of electron (e^−^) transfer and associated chemical reactions.

The architecture of the ETC is illustrated in Figure 1. In the RGC, the efficiency of this system is a critical survival determinant. Any compromise in the ETC, measured as OCR, results in an immediate bioenergetic deficit [33].

Extensive research has identified a specific defect in Complex I (NADH:ubiquinone oxidoreductase) as a hallmark of glaucomatous bioenergetics [34,35,36]. Electrons mainly enter the respiratory chain via the Complex I pathway. Systemic tissues from POAG patients consistently demonstrate a specific Complex I defect, characterized by impaired NADH-linked respiration [10,34]. This defect is often driven by somatic mutations in the mitochondrial genome. Specifically, sequencing studies by Sundaresan et al. and Abu-Amero et al. have identified a high prevalence of transition and transversion mutations in the *MT-ND1* and *MT-ND5* genes, a recognized mutational hotspot in POAG, both of which encode critical subunits of Complex I [23,35,36]. Vallbona-Garcia et al. [37] have further elucidated the genetic basis of this dysfunction. Their studies identified that variants in the mitochondrial D-loop, specifically within the 7S DNA sub-region, are significantly enriched in patients with HTG. The D-loop is the control region for mtDNA replication and transcription. Variants in this region can impair the replication machinery, leading to the reduced mtDNA copy numbers observed in the blood of HTG patients [11,37].

Complex I dysfunction impairs electron transport, limits ATP production, and increases electron leakage to generate superoxide anions that drive oxidative stress [20]. This intrinsic defect in the primary step of oxidative phosphorylation underlies the clinically observed reduction in OCR [9].

### 3.3. The NAD^+^ Depletion Axis and SARM1-Mediated Degeneration

A critical downstream consequence of mitochondrial dysfunction is the depletion of NAD^+^, an essential coenzyme for redox reactions and a signaling metabolite for axonal survival [10,38]. In healthy axons, NAD^+^ levels are maintained by the enzyme nicotinamide mononucleotide adenylyltransferase 2 (NMNAT2), which converts nicotinamide mononucleotide (NMN) into NAD^+^. NMNAT2 is labile and must be continuously transported from the soma to the axon [39,40].

**Figure 2:**
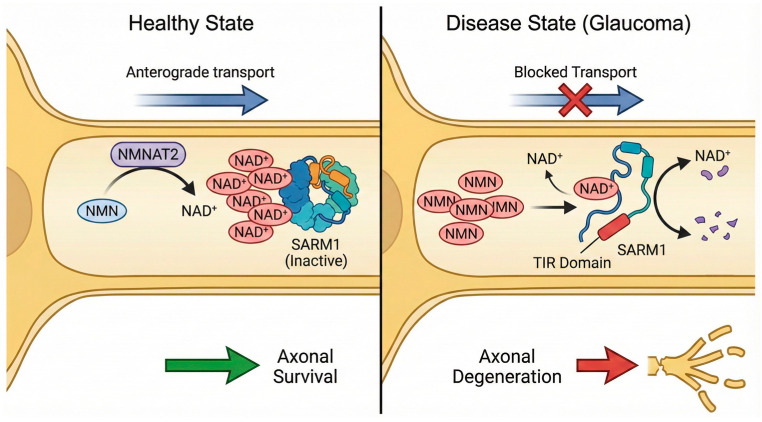
The SARM1-mediated axonal degeneration pathway. Under homeostatic conditions (**Left**), the enzyme NMNAT2 is transported to the axon, converting NMN to NAD^+^. High axonal levels of NAD^+^ bind to the regulatory ARM domain of SARM1, maintaining it in an auto-inhibited state. In glaucoma (**Right**), bioenergetic failure or mechanical stress impairs anterograde transport, depleting local NMNAT2. This results in the accumulation of NMN and a reduction in NAD^+^, triggering a conformational change in SARM1. The activated TIR domain of SARM1 rapidly hydrolyzes the remaining NAD^+^, leading to metabolic collapse and irreversible axonal fragmentation. Colored shapes are utilized to visually differentiate specific metabolites, proteins, and protein domains. The blue arrow represents anterograde transport and is crossed out in red to indicate a blockade. The green and red arrows denote the overall physiological outcomes of axonal survival and degeneration, and black arrows indicate biochemical conversions and molecular interactions.

In glaucoma, transport failure or bioenergetic stress leads to a local depletion of NMNAT2. This results in two distinct metabolic failures comprising a decrease in axonal NAD^+^ and an accumulation of its precursor NMN [40]. This shift in the NMN/NAD^+^ ratio acts as a molecular switch for SARM1, which serves as the central executioner of Wallerian degeneration [17]. The molecular mechanism of this pathway is illustrated in Figure 2.

SARM1 functions as a metabolic sensor. Under normal conditions, high NAD^+^ levels bind to the SARM1 regulatory ARM domain and maintain the protein in an auto-inhibited octameric state [41]. However, recent structural analyses utilizing cryo-electron microscopy have demonstrated that an increase in NMN relative to NAD^+^ allows NMN to take the place of NAD^+^ at the ARM domain allosteric pocket [17]. Such binding forces a conformational realignment that frees the TIR domain.

Once activated, SARM1 functions as a potent NADase, rapidly hydrolyzing the remaining NAD^+^ in the axon. This precipitates an irreversible metabolic collapse, calcium influx, and structural fragmentation of the axon [17]. Animal models have shown that SARM1 knockout mice are robustly protected against RGC loss in a chronic ocular hypertension model [42]. The study showed that loss of SARM1 reduced RGC death significantly compared to wild-type controls, even in the presence of elevated IOP. Furthermore, Tribble et al. identified NMNAT2 as a pharmacological target, showing that small molecule activators of NMNAT2 can drive neuronal NAD^+^ production and provide neuroprotection [43]. Table 1 summarizes key genetic and pharmacologic manipulations of the SARM1/NMNAT2 axis in experimental models.

### 3.4. Oxidative Stress and mtDNA Damage

The ocular environment is uniquely predisposed to oxidative damage. In glaucoma, the leakiness of the dysfunctional ETC exacerbates ROS generation, overwhelming antioxidant defenses like superoxide dismutase 2 [20]. This leads to the accumulation of 8-OHdG, a marker of oxidative DNA damage [31].

Studies have consistently found significantly elevated levels of 8-OHdG in the aqueous humor and serum of glaucoma patients compared to cataract controls [44]. Furthermore, high levels of this DNA damage marker correlate with the rate of visual field progression in NTG, validating oxidative stress not just as a byproduct, but as a driver of disease worsening [45]. At the genomic level, this manifests as an enrichment of transversions and specific large-scale deletions in the TM, creating a feed-forward loop of mitochondrial decline [22,46].

## 4. Peripheral Blood Mononuclear Cells (PBMCs) as Systemic Bioenergetic Windows

Due to the inaccessibility of live retinal tissue, PBMCs serve as an accessible systemic proxy. Their metabolic profile functionally mirrors RGCs through a triad of factors:

- Shared systemic mtDNA variants establishing a baseline bioenergetic vulnerability [47];
- Simultaneous exposure to circulating insults such as ROS and cytokines because the ONH lacks a stringent blood–retinal barrier (BRB) [48,49];
- Exceptionally high metabolic demands that render both cell types selectively vulnerable to systemic Complex I and NAD^+^ deficits [9].

Consequently, PBMCs sensitively reflect the glaucomatous neurovascular microenvironment. Validating this model, recent proteomic profiling of PBMCs in POAG subjects has identified significant alterations in pathways regulating immune function, autophagy, and mitochondrial metabolism, mirroring the neurodegenerative events in the eye [50].

### 4.1. PBMC Oxygen Consumption Rate (OCR) as a Biomarker

The utility of PBMCs in glaucoma management has been validated by Petriti et al. utilizing extracellular flux analysis to profile mitochondrial respiration in glaucoma patients [9].

The findings indicated the following:

- Reduced Respiration: Patients with POAG exhibited significantly lower basal and maximal OCR relative to healthy age-matched individuals [9].
- Correlation with Progression: Lower PBMC OCR correlated with accelerated visual field loss and retinal nerve fiber layer (RNFL) thinning, independent of IOP. This correlation persisted even patients with well-managed IOP [9].

These results imply that systemic bioenergetic capacity acts as a resistance factor. Currently, impaired PBMC OCR functions primarily as a trait marker identifying baseline intrinsic bioenergetic fragility, which lowers the threshold for RGC damage under localized stress such as elevated IOP [9,33,50]. Further longitudinal studies are required to determine if acute metabolic fluctuations can also serve as a state marker of real-time disease progression, which will dictate whether profiling is best suited for initial risk stratification or longitudinal monitoring.

### 4.2. Systemic NAD^+^ Depletion

Paralleling the respirometric data, Petriti et al. [9] also found that total NAD^+^ levels were substantially lower in PBMCs from POAG subjects relative to those of healthy counterparts. Furthermore, systemic NAD^+^ levels correlated positively with OCR, linking the specific molecular deficit of NAD^+^ depletion to the functional output of respiration [51]. This systemic depletion mirrors the axonal deficits described in the SARM1 pathway, suggesting a generalized failure of NAD^+^ biosynthesis or accelerated consumption in glaucoma patients [39,42].

### 4.3. Proteomic Alterations: The Bioenergetic Blockade of Autophagy

Proteomic profiling indicates that the bioenergetic crisis, specifically the combined effects of the systemic Complex I defect and NAD^+^ depletion, is closely coupled with a widespread impairment in protein clearance mechanisms [50,52]. Mechanistically, intrinsic mitochondrial dysfunction acts as the primary upstream trigger. Basal Complex I dysfunction driven by somatic mutations such as those in the *MT-ND5* gene limits ATP production and generates ROS, which subsequently signal for autophagic initiation through the upregulation of Beclin-1 [23,50].

However, impaired mitophagy manifests as a downstream consequence. Because the completion of autophagic and mitophagic flux is a highly energy-dependent process, the concurrent depletion of ATP and NAD^+^ deprives the cells of the requisite energetic substrates to execute the final stages of vesicle maturation and lysosomal degradation [53,54]. Giammaria et al. characterized this phenotype of abortive autophagy within human PBMCs in which the cellular machinery attempts to initiate autophagic flux, as evidenced by the upregulation of initiation factors, yet fails to complete the process due to downstream defects in vesicle maturation, such as the downregulation of Atg9A [50].

This bioenergetic dissociation between initiation and clearance parallels the pathology identified in the DBA/2J murine model. Hirt et al. demonstrated that although stress induces autophagic signaling, as evidenced by upregulated LC3-II, the degradative phase remains compromised due to a critical deficiency in lysosomal capacity marked by downregulated LAMP1 [55]. A detailed inventory of the specific upregulated and downregulated proteomic targets identified in these investigations is presented in Table 2.

The concurrent upregulation of Beclin-1 and accumulation of p62 indicate a specific failure in mitophagy, defined as the clearance of damaged mitochondria [50,55]. When mitochondria become defective, they must be degraded to prevent ROS leakage. In glaucoma patients, this mitophagic flux is impaired, leading to the retention of damaged organelles. The consequent accumulation of p62 alongside oxidative stress exacerbates the bioenergetic deficit, worsens NAD^+^ depletion, and instigates sterile inflammatory pathways via the NLRP3 inflammasome [55,56]. Thus, while mitophagic failure is initially a downstream consequence of energy starvation, the retention of these leaking organelles transforms the pathology into a self-sustaining feed-forward loop.

### 4.4. Limitations of the Systemic Proxy: Tissue Decoupling and Confounders

While PBMCs offer an accessible window into systemic metabolism, relying on this proxy presents inherent limitations that may decouple systemic readouts from localized retinal stress:

- Tissue and Cell-Type Specificity: The retinal metabolic environment is characterized by the concurrent utilization of aerobic glycolysis, commonly referred to as the Warburg effect, and oxidative phosphorylation [57]. Furthermore, the ocular microenvironment exhibits significant metabolic heterogeneity. While circulating PBMCs reflect a shared basal vulnerability, they cannot capture the distinct localized demands or cross-talk of specific ocular populations. For example, RGCs requiring massive ATP for unmyelinated axonal conduction, TM cells requiring energy for contractile tone and extracellular matrix remodeling, or LC cells managing structural support.
- Systemic Confounders and Comorbidities: As immune cells, PBMCs are sensitive to systemic physiological changes. Baseline bioenergetics can be confounded by comorbidities prevalent in aging populations, such as diabetes mellitus and cardiovascular disease, which inherently disrupt mitochondrial function [58,59].
- Medication Interference: Routine pharmacological interventions directly alter mitochondrial respiration, including statins, systemic corticosteroids, and metformin (a known Complex I inhibitor), skewing PBMC OCR independently of glaucoma status [60,61].
- Inflammatory States: Acute infections or chronic systemic autoimmune conditions can force a metabolic shift toward glycolysis to support immune activation, artificially depressing OCR and depleting NAD^+^ [62].

## 5. Metabolic Phenotypes in Glaucoma Subtypes

### 5.1. Normal Tension Glaucoma (NTG)

Respirometric data position NTG at the most compromised end of the metabolic spectrum, with significantly lower mitochondrial function than HTG patients and controls [9,46]. In NTG, mitochondria are intrinsically inefficient, meaning they possess minimal SRC [63,64]. While vascular dysregulation and systemic hypotension are well-established parallel drivers in NTG, this profound bioenergetic fragility elucidates why these patients often progress despite aggressive IOP reduction. Even normal physiological IOP acts as a sufficient stressor to deplete these diminished reserves and trigger degeneration when interacting with these concurrent susceptibilities.

### 5.2. High Tension Glaucoma (HTG)

Conversely, HTG is hypothesized to represent a secondary metabolic insufficiency. While impaired relative to controls, mitochondrial function in HTG is generally superior to NTG. Although elevated IOP-induced mechanical stress is the primary driver, HTG patients often harbor reduced peripheral mtDNA copy numbers [9,11,30]. This suggests that while mechanical strain initiates damage, an underlying baseline metabolic vulnerability persists and may dictate progression rates.

### 5.3. Exfoliation Glaucoma (XFG)

XFG, driven by variants in the LOXL1 gene, is an aggressive and recalcitrant form of the disease [65,66]. It represents a systemic elastotic disorder with severe bioenergetic consequences. Research by Venkatesan et al. has elucidated the mechanism by which pathogenic LOXL1 variants lead to protein misfolding and aggregation in Tenon’s capsule fibroblasts and ocular tissues [67]. These aggregates induce endoplasmic reticulum stress and directly impair mitochondrial function. Importantly, this pathology also involves profound microtubule defects, providing a mechanistic intersection with the broader bioenergetic crisis: disrupted microtubule networks stall both the axoplasmic transport of mitochondria and the trafficking of mitophagy machinery, mirroring the abortive autophagy bottlenecks previously described. Consequently, XFG fibroblasts exhibit significantly reduced ATP production and SRC [67,68]. This systemic bioenergetic failure compounds the primary mechanical insult of TM obstruction, and the convergence of mechanical blockage, cytoskeletal breakdown, and cellular energy failure drives the rapid progression characteristic of XFG.

## 6. Diagnostic and Prognostic Indices

The translation of these biological insights into clinical practice is underway through the development of novel diagnostic indices that quantify metabolic risk.

### 6.1. Metabolic Risk Score (MRS)

While the polygenic risk score (PRS) defined by Craig et al. effectively categorizes genetic susceptibility, Li et al. integrated these data with metabolomics to refine risk stratification [69,70]. They identified a metabolomic signature associated with resilience, characterized by elevated systemic levels of pyruvate, lactate, and citrate which informed an MRS.

Stratification analysis revealed that a favorable MRS significantly attenuated the odds of developing glaucoma, even among individuals with the highest genetic burden, whereas the convergence of peak genetic liability with peak metabolic risk resulted in markedly elevated susceptibility. This interaction underscores the capacity of serum metabolite profiling to distinguish genetically susceptible patients who are metabolically protected from those with combined burden [69,70].

To translate these findings into real-world practice, future risk calculators must evolve beyond traditional parameters (IOP, central corneal thickness, family history). By integrating static genetic susceptibility (PRS) with dynamic metabolomic signatures (MRS) and standard clinical factors, clinicians can develop comprehensive, individualized risk profiles to better predict IOP-independent progression.

### 6.2. Flavoprotein Fluorescence (FPF)

To evaluate intraocular mitochondrial function directly, FPF imaging has been developed [71]. This non-invasive technique exploits the autofluorescence of oxidized flavin adenine dinucleotide (FAD) in mitochondrial lipoamide dehydrogenase and electron transfer flavoprotein [72]. When the ETC is functioning efficiently, FAD is largely reduced to FADH2 and is non-fluorescent. However, when the ETC is stalled or stressed, resulting in dysfunction, the pool of oxidized FAD increases and emits a green fluorescence (520–540 nm) upon excitation with blue light [73].

Clinical studies demonstrate the most robust signal changes occur specifically within the ONH and peripapillary retina, where FPF intensity is significantly higher in glaucoma patients than controls [67,74]. Some evidence suggests that elevated FPF is detectable in glaucoma suspects and OHT patients before significant structural thinning occurs on OCT, potentially serving as an early biomarker of metabolic stress and impending neurodegeneration [67,75,76]. However, universally accepted clinical threshold values for FPF have not yet been established. Furthermore, some findings remain equivocal. For instance, Caro et al. observed no significant differences in FPF values in the ONH between healthy, suspect, and POAG cohorts [75], underscoring the need for larger longitudinal studies to define reliable diagnostic cut-offs before FPF can routinely guide treatment decisions.

## 7. Targeting Mitochondrial Resilience: Therapeutic Application

The identification of NAD^+^ depletion, Complex I defects, and SARM1 activation has established the foundation of metabolic neuroprotection. The therapeutic goal is shifting from solely lowering IOP to enhancing the SRC of the RGC.

### 7.1. Nicotinamide (NAM)

NAM represents the primary metabolic agent. As a precursor to NAD^+^, high-dose supplementation replenishes the depleting NAD^+^ levels, preventing the decrease in NMN/NAD^+^ ratio that induces SARM1 activation [17]. Additionally, NAD^+^ boosts Complex I activity and supports DNA repair via PARP enzymes [77].

A randomized Phase 2 study demonstrated that a combination of NAM and pyruvate resulted in a marked improvement in visual function manifesting as short-term visual field recovery in glaucoma patients. This functional gain is rarely seen with IOP lowering alone [78]. Furthermore, Hui et al. demonstrated a 14.8% improvement in the physiological performance of the inner retina, quantified via the amplitude of the photopic negative response following NAM supplementation, representing a degree of recovery rarely achieved with traditional IOP-lowering management alone [79].

### 7.2. The Nicotinamide in Glaucoma (NAMinG) Phase III Trial and Safety Profile in Elderly Populations

The ongoing NAMinG Phase III trial evaluates high-dose oral NAM (3.0 g/day for 27 months) to determine if systemic metabolic support can slow visual field deterioration rates in patients with OAG who are already receiving standard IOP-lowering therapy [80]. Pending favorable trial outcomes, NAM would become the first validated non-IOP lowering neuroprotective treatment for glaucoma.

However, translating high-dose NAM therapy to routine clinical practice requires rigorous safety monitoring. Longitudinal exposure carries documented risks of drug-induced liver injury and elevated serum transaminases [81]. This hepatotoxic potential is particularly critical for elderly glaucoma patients who frequently exhibit age-related declines in hepatic clearance and face adverse polypharmacy interactions from multiple comorbidities. Therefore, the American Glaucoma Society and the American Academy of Ophthalmology currently caution against unsupervised high-dose NAM supplementation outside of clinical trials [82]. Future implementation will necessitate a collaborative hepatic monitoring framework between ophthalmologists and primary care physicians.

### 7.3. Pyruvate

Pyruvate supplementation addresses the bioenergetic deficit from a different angle. As a key substrate for the Krebs cycle, pyruvate bypasses the rate-limiting steps of glycolysis and directly drives mitochondrial respiration. Li et al. confirmed that high systemic pyruvate levels confer resilience to glaucoma. Animal models have shown that dietary pyruvate protects RGCs from IOP-induced death and increases the survival of cells with mitochondrial defects [69].

### 7.4. HDAP2

HDAP2, exclusively found within the inner mitochondrial membrane, is a high-density aromatic peptide that stabilizes membranes by binding to cardiolipin. In glaucoma, oxidative stress triggers cardiolipin peroxidation, resulting in the destruction of membrane integrity and the release of cytochrome c to initiate cell death [83]. In experiments using the DBA/2J mouse model of spontaneous glaucoma, MacNeil et al. demonstrated that systemic HDAP2 administration improved RGC survival by approximately 49% relative to the control group. Furthermore, the peptide was effective in preserving both axons and RGCs across a spectrum of pressure exposures, effectively increasing the tissue’s resilience to IOP [84].

Translating peptide therapeutics like HDAP2 from murine models to humans presents substantial delivery difficulties [84]. Systemic delivery is severely restricted by the BRB and inherent proteolytic degradation [85]. Conversely, localized intravitreal injection bypasses the BRB but introduces distinct translational challenges. The murine pharmacokinetics do not linearly translate to the substantially larger human vitreous volume. Maintaining therapeutic RGC concentrations will likely necessitate advanced, sustained-release formulations to avoid frequent invasive injections.

### 7.5. Coenzyme Q_10_ (CoQ_10_) and Ubiquinol

CoQ_10_ acts as an essential electron carrier between Complex I/II and Complex III, bypassing the specific Complex I defects observed in glaucoma PBMCs while neutralizing ROS [9]. Mechanistically, CoQ_10_ stabilizes the Bax/Bcl-2 axis; in models of retinal ischemia, it upregulates anti-apoptotic Bcl-xL and downregulates pro-apoptotic Bax, preventing the opening of the mitochondrial permeability transition pore [86]. Furthermore, CoQ_10_ mitigates neuroinflammation by reducing GFAP expression in reactive astrocytes and dampening the release of pro-inflammatory cytokines such as TNF-α and IL-1β [86,87]. Moreover, Dogan et al. reported that POAG patients treated with topical CoQ_10_/vitamin E for 12 months showed significantly decreased visual evoked potential P100 implicit times and increased amplitudes compared to controls, suggesting protection of RNFL thickness [88].

### 7.6. Metformin

While traditionally an anti-diabetic agent, metformin has emerged as a therapeutic candidate based on its neuroprotective and anti-fibrotic properties observed in preclinical models [89]. Epidemiologically, retrospective cohort studies, such as those by Sidhu et al., have found metformin use to be associated with a reduction in POAG risk, whereas other diabetic medications were associated with increased risk [90]. Mechanistically, metformin activates the primary cellular energy sensor AMP-activated protein kinase (AMPK). By mildly inhibiting Complex I and increasing the AMP/ATP ratio, metformin triggers AMPK to inhibit mTOR, thereby releasing the brake on autophagy. This restores autophagic flux, upregulates mitophagy receptors such as parkin and optineurin, and facilitates the removal of dysfunctional organelles alongside accumulated proteins like p62 [91,92]. Additionally, *in vitro* studies suggest metformin targets the trabecular meshwork by modulating the integrin/Rho-associated kinase pathway to rearrange the cytoskeleton and reduce fibrotic markers, thereby alleviating outflow resistance [89]. Satriano et al. demonstrated a hormetic effect in murine models in which low-dose metformin (10 mg/kg) significantly prevented RGC loss, whereas high doses were ineffective, highlighting the importance of optimal dosing windows [92].

However, these promising mechanistic and observational findings lack validation from completed randomized controlled trials in human cohorts. While a trial investigating metformin’s potential to preserve RNFL thickness in non-diabetic patients is ongoing, its application as a glaucoma therapeutic remains investigational until prospective clinical endpoints are met [93].

### 7.7. Precision Metabolic Therapy Across Glaucoma Subtypes and Trial Stratification

The heterogeneity of glaucoma necessitates tailored metabolic interventions. In NTG, the primary deficit is intrinsic bioenergetic fragility and diminished SRC, and the therapeutic priority is maximizing ATP output and electron transport efficiency. An optimal therapeutic regimen for NTG would primarily feature NAM, pyruvate, and CoQ_10_ to directly fuel respiration and bypass Complex I defects [33,78]. In contrast, XFG represents a dual pathology where LOXL1-driven protein misfolding induces severe endoplasmic reticulum stress and secondary mitochondrial collapse [94]. For XFG, energetic supplementation alone is insufficient without concurrently addressing the proteasome impairment [95]. Therefore, hypothetically, a targeted XFG metabolic intervention might benefit from incorporating robust autophagy inducers. Based on the aforementioned preclinical models, agents such as metformin, which enhance AMPK and increase autophagic flux, could theoretically facilitate the clearance of toxic LOXL1 aggregates [92]. However, rigorously designed clinical trials are required to determine whether such mechanistically targeted approaches can safely and effectively yield measurable visual benefits in patients with exfoliation syndrome.

Future clinical trials must move beyond reliance solely on IOP criteria and incorporate active metabolic stratification to prevent diluting positive treatment effects across heterogeneous cohorts:

- NAM Trials: Enrich cohorts with patients exhibiting significantly reduced basal PBMC OCR and profound systemic NAD^+^ depletion.
- Pyruvate Trials: Target individuals identified via MRS profiling as lacking protective circulating pyruvate and citrate.
- Metformin/AMPK Trials: Prioritize patients with XFG or biomarkers of severe proteostatic failure to restore autophagic flux.

## 8. Limitations

While the metabolic theory of glaucoma offers a compelling framework, several limitations remain.

First, as detailed in Section 4.4, using PBMCs as a proxy is complicated by tissue-specific metabolic decoupling and systemic confounders. Furthermore, translating therapies involve significant safety and bioavailability hurdles. High-dose NAM exceeds the standard dietary allowance and increases risks of hepatotoxicity [81], demanding rigorous monitoring in elderly patients. Similarly, peptide therapeutics like HDAP2 face pharmacokinetic barriers, including BRB impermeability and human vitreous scaling challenges.

Second, phenotypic heterogeneity challenges standardized treatment. Patients exhibit differing metabolic vulnerabilities [69], meaning broad interventions like mechanical factors or other genetic risks may yield variable results without precise clinical stratification tools.

Third, accelerated murine models like DBA/2J do not perfectly recapitulate the decades-long chronic progression of human glaucoma, and may be more responsive to acute metabolic rescue [55,84].

Finally, a significant translational gap exists between robust preclinical signals, such as the hormetic effects of metformin, and proven human efficacy, necessitating large-scale randomized controlled trials. To overcome these hurdles and translate the mitochondrial theory into clinical care, future research must prioritize:

- Multicenter validation of PBMC OCR assays and standardized NAD^+^ measurement platforms;
- Harmonization of in vivo imaging like FPF to establish universal clinical thresholds;
- Biomarker-guided trial designs, using MRS, OCR, or NAD^+^ levels to actively stratify cohorts and ensure targeted, precision neuroprotection.

## 9. Conclusions

The evidence synthesized in this review compels a re-evaluation of glaucoma management, expanding from purely IOP-lowering strategies to incorporate metabolic neuroprotection. While IOP remains the primary adjustable risk factor, the bioenergetic resilience of the RGC and the systemic physiology of the patient determine the threshold for blindness.

**Figure 3:**
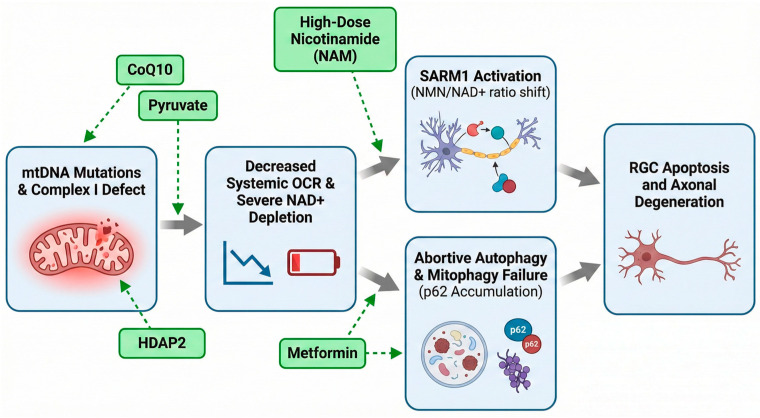
Integrated Model of Glaucomatous Metabolic Vulnerability and Therapeutic Intervention. The sequential pathogenesis from upstream mitochondrial dysfunction to downstream retinal ganglion cell (RGC) death, overlaid with targeted metabolic therapies. Blue boxes represent the sequential stages of disease pathogenesis, green boxes indicate targeted therapeutic interventions, grey arrows denote the progression of pathological events, and dashed green arrows illustrate the specific targets of the applied metabolic therapies. mtDNA, mitochondrial DNA; CoQ_10_, coenzyme Q_10_; OCR, oxygen consumption rate.

As summarized in Figure 3, somatic mtDNA mutations (e.g., *MT-ND5*) induce a systemic Complex I defect, creating metabolic vulnerability. Under IOP-induced stress, this vulnerability manifests as NAD^+^ depletion and NMN accumulation, triggering SARM1-mediated axonal degeneration. Systemically mirrored in PBMCs via reduced OCR, NAD^+^ depletion, and autophagic blockade characterized by p62 accumulation, these biomarkers correlate strongly with vision decline.

Consequently, glaucoma management is evolving towards a dual strategy. This approach involves lowering mechanical stress while simultaneously elevating the bioenergetic threshold through agents like nicotinamide, pyruvate, HDAP2, CoQ_10_, and metformin. However, translating these findings requires caution due to PBMC proxy limitations and disease heterogeneity. Further research relies on developing precision stratification tools to identify the specific patient subsets most likely to benefit from this bioenergetic framework.

## Figures and Tables

**Table 1:** Key Genetic and Pharmacologic Manipulations of the SARM1/NMNAT2 Axis.

Target	Manipulation Type	Experimental Model	Key Findings	Reference
SARM1	Genetic Knockout	Chronic ocular hypertension (Mice)	Loss of SARM1 robustly protects against RGC ^1^ loss and reduces cell death significantly compared to wild-type controls, even in the presence of elevated IOP ^2^.	Zeng et al. [42]
NMNAT2	Pharmacologic Activation	Preclinical neurodegeneration models	Small molecule activators specifically target NMNAT2 to drive neuronal NAD^+^ production, offering robust neuroprotection against axonal degeneration.	Tribble et al. [43]
	Gene Therapy (Overexpression)	Glaucomatous RGC models	Counteracts the disease-state downregulation of NMNAT2; RGC-specific gene therapy rescues neurodegeneration and preserves visual function.	Fang et al. [39]

^1^ RGC, Retinal ganglion cell; ^2^ IOP, Intraocular pressure.

**Table 2:** Key Proteomic Markers of Autophagic and Proteolytic Dysregulation in Glaucoma PBMCs.

Methodology	Key Proteomic Findings	Implication in Pathogenesis	Study
Shotgun Proteomics (Human PBMC ^1^)	Upregulated: Beclin-1, Bcl2 ^2^, NAD kinase	Abortive Autophagy: The upregulation of initiation markers (Beclin-1) is futile due to downstream defects in vesicle maturation (Atg9A), preventing organelle clearance.	Giammaria et al. [50]
	Downregulated: Atg9A ^3^, PSMA3/4 ^4^, Atlastin-2		
Immunofluorescence (DBA/2J Mice)	Upregulated: LC3-II ^5^, p62	Lysosomal Exhaustion: A depletion of lysosomes (LAMP1) creates a bottleneck, stalling the flux and causing the pathological accumulation of p62 and debris.	Hirt et al. [55]
	Downregulated: LAMP1 ^6^		

^1^ PBMC, Peripheral blood mononuclear cell; ^2^ Bcl2, B-cell lymphoma 2; ^3^ Atg9A, Autophagy-related protein 9A; ^4^ PSMA3/4, Proteasome 20S subunit alpha 3/beta 4; ^5^ LC3-II, Microtubule-associated protein 1A/1B-light chain 3-II; ^6^ LAMP1, Lysosomal-associated membrane protein 1.

## Data Availability

The original contributions presented in this study are included in the article. Further inquiries can be directed to the corresponding authors.
